# WORKING THROUGH IT: EMPLOYMENT DISCRIMINATION, LABOR FORCE OUTCOMES, AND MENTAL HEALTH AMONG OLDER BLACK AMERICANS

**DOI:** 10.1093/geroni/igad104.1225

**Published:** 2023-12-21

**Authors:** Lauren Brown, Leah Abrams, Yuan Zhang

**Affiliations:** University of Southern California, Los Angeles, California, United States; Tufts University, Medford, Massachusetts, United States; Columbia University, New York City, New York, United States

## Abstract

We examine lifetime experiences of employment discrimination as one pathway by which racism impacts Black older adults’ well-being. We use data from the Health and Retirement Study’s (HRS) leave behind questionnaire to characterize lifetime experiences of being unfairly fired, not hired, or not promoted among Black older adults (N=2,948) and test associations of employment discrimination with labor force status at age 62, job satisfaction among those working, and depressive symptoms. Employment discrimination was commonly reported by Black older adults, especially among men and those with any college education. Employment discrimination was not associated with employment status but was associated with job dissatisfaction (OR=2.00, p=0.001) and depressive symptoms (Beta=0.34, p< 0.001). Findings demonstrate the detrimental impact of employment discrimination on Black older adults’ work experiences and mental health, while highlighting the limitations of HRS’s discrimination items. This research points to racism in the workplace as an obstacle to healthy aging for Black adults in America.

